# 311 service requests as indicators of neighborhood distress and opioid use disorder

**DOI:** 10.1038/s41598-020-76685-z

**Published:** 2020-11-11

**Authors:** Yuchen Li, Ayaz Hyder, Lauren T. Southerland, Gretchen Hammond, Adam Porr, Harvey J. Miller

**Affiliations:** 1 Department of Geography, The Ohio State University, Columbus, OH USA; 2 Center for Urban and Regional Analysis, The Ohio State University, Columbus, OH USA; 3 College of Public Health, The Ohio State University, Columbus, OH USA; 4 Wexner Medical Center, The Ohio State University, Columbus, OH USA; 5 Mighty Crow Media, Columbus, OH USA

**Keywords:** Public health, Epidemiology, Risk factors

## Abstract

Opioid use disorder and overdose deaths is a public health crisis in the United States, and there is increasing recognition that its etiology is rooted in part by social determinants such as poverty, isolation and social upheaval. Limiting research and policy interventions is the low temporal and spatial resolution of publicly available administrative data such as census data. We explore the use of municipal service requests (also known as “311” requests) as high resolution spatial and temporal indicators of neighborhood social distress and opioid misuse. We analyze the spatial associations between georeferenced opioid overdose event (OOE) data from emergency medical service responders and 311 service request data from the City of Columbus, OH, USA for the time period 2008–2017. We find 10 out of 21 types of 311 requests spatially associate with OOEs and also characterize neighborhoods with lower socio-economic status in the city, both consistently over time. We also demonstrate that the 311 indicators are capable of predicting OOE hotspots at the neighborhood-level: our results show code violation, public health, and street lighting were the top three accurate predictors with predictive accuracy as 0.92, 0.89 and 0.83, respectively. Since 311 requests are publicly available with high spatial and temporal resolution, they can be effective as opioid overdose surveillance indicators for basic research and applied policy.

## Introduction

In the United States, the rise in rates of substance use disorder is a public health crisis^1^, reaching epidemic levels^2^. Columbus, Ohio is at the heart of the opioid epidemic: Ohio continues to be a center of the opioid epidemic, ranking in the top five states for opioid-related overdose deaths as reported by the National Institute on Drug Abuse^3^. Opioid use disorder is the main cause of death among drug users^4^. Opioid supply and availability is a factor^5^, but it is not the only one. The national overdose death rate surged 38% between 2012 and 2015, despite a 13% drop in the outpatient opioid analgesic prescriptions during those years^6^. There has also been a shift to illicit opioids like heroin and fentanyl^7^; however, the prescription and illicit sources still do not fully explain the dynamic and spatial heterogeneity of the opioid epidemic in regions and communities^8,9^.

There is increasing recognition of the role that social and neighborhood determinants play in health outcomes^10,11^. This places attention on the health effects of upstream social factors such as economic, education, and demographic that shape downstream factors such as behavior, economic stability, stress levels, support networks, neighborhood and physical environment, and access to healthy food and health care^10^. Recent analyses have documented the social and economic factors may be related to the increase of self-destructive health behaviors such as opioid misuse leading to addiction^12–14^. Studies shown in much of the country, the counties with the lowest levels of social capital have the highest overdose rates, suggesting the role of community fragility in the drug overdose epidemic^15^. Residential mobility studies suggest that drug misuse improves when people move to a community with less social and economic disadvantage^16^. There is increasing recognition that the opioid crisis is fueled by economic and social upheaval; its etiology rooted in concentrated disadvantages, isolation and social upheaval^6^.

Overdose-related events are spatially dynamic, spreading differentially through regions and neighborhoods^17–19^. Accurate surveillance is vital for detecting outbreaks and for guiding interventions to mitigate the opioid epidemic^20^. Understanding and planning for opioid crisis interventions can benefit from high-resolution spatial and temporal data on social and neighborhood attributes that associate with overdose incidents. However, much of the social and economic data available to researchers and planners is updated at infrequent intervals (in the case of US Census data, 5–10 years) and spatially aggregated (census zones, ZIP codes, jurisdictions). These data are also impacted by the shape and size of the boundary of aggregation, a version of the ecological fallacy^21^ known as the modifiable area unit problem^22^. Easily available, high spatial and temporal resolution data that indicates the opioid crisis due to social and neighborhood distress can help guide basic research and policy interventions^20,23^.

The digitization of urban services provides new sources of data that can resolve the weaknesses of traditional administrative data in understanding and mitigating the opioid crisis at the community level. 311 data comprise non-emergency service requests by residents, received traditionally via phone but now commonly by email or webforms. 311 requests reflect a wide range of neighborhood issues is such as streetlight repair, abandoned vehicles, code violations and noise, that reflect actual and perceived disorder, distress and neglect. These are public data that are frequently updated (in many cases, daily) and have high spatial resolution (latitude and longitude). In contrast, 911 emergency request data are not widely available due to privacy concerns; they also point to the outcome (an overdose event) rather than the antecedents (social and neighborhood distress).

We examine the use of 311 service requests as indicators of neighborhood distress and opioid overdose incidents. We identify 311 request types that can serve as robust surveillance indicators for opioid use disorder based on three criteria: (1) spatial association with individual-level opioid overdose events (OOEs); (2) characterize neighborhoods with apparent conditions of socioeconomic distress, and; (3) stability of these relationships with respect to time. We also demonstrate their use in predicting OOE hotspots at the neighborhood level.

Using address-level opioid overdose event (OOE) data from emergency medical services and 311 request data for the years 2008–2017 in Columbus, Ohio, USA, we use an inhomogeneous cross-*K*-function to discover the spatial association between georeferenced individual-level OOEs and 311 service requests types. We also identify the types of 311 requests that characterize neighborhoods with socioeconomic distress using k-means clustering of 311 data and an analysis of variance (ANOVA) test for differences among neighborhood clusters based on socioeconomic data from the US Census. We assess the stability of the spatial associations between 311 requests and OOEs by applying same inhomogeneous cross-*K*-function at a quarterly rather than annual resolution. We further identify robust indicators as the 311 request types that have persistent relationships with OOEs and neighborhood distress over time. We use an individual level predictive model and evaluate the performance of the robust 311 indicators in predicting overdose hotspots.

The results from this study support the view that opioid crisis is rooted in social and neighborhood distress. We show such spatial characteristics can be used along with 311 data itself to predict the trends of opioid overdose hotspots when OOEs data is not available. Since 311 requests are publicly available and with high spatial and temporal resolution, they can be effective as opioid overdose surveillance indicators for basic research and applied policy.

## Results

### Spatial associations between OOEs and 311 service calls

The inhomogeneous cross-*K*-function identifies the spatial dependencies between 311 calls and OOEs. For each distance, if the L-function (a linearly transformed *K*-function) goes beyond the upper bound or below the lower bound of the complete spatial randomness (CSR) envelope, we conclude the OOEs are spatially clustered/dispersed with specified 311 calls correspondingly. Otherwise the spatial relationship is random.

**Figure 1:**
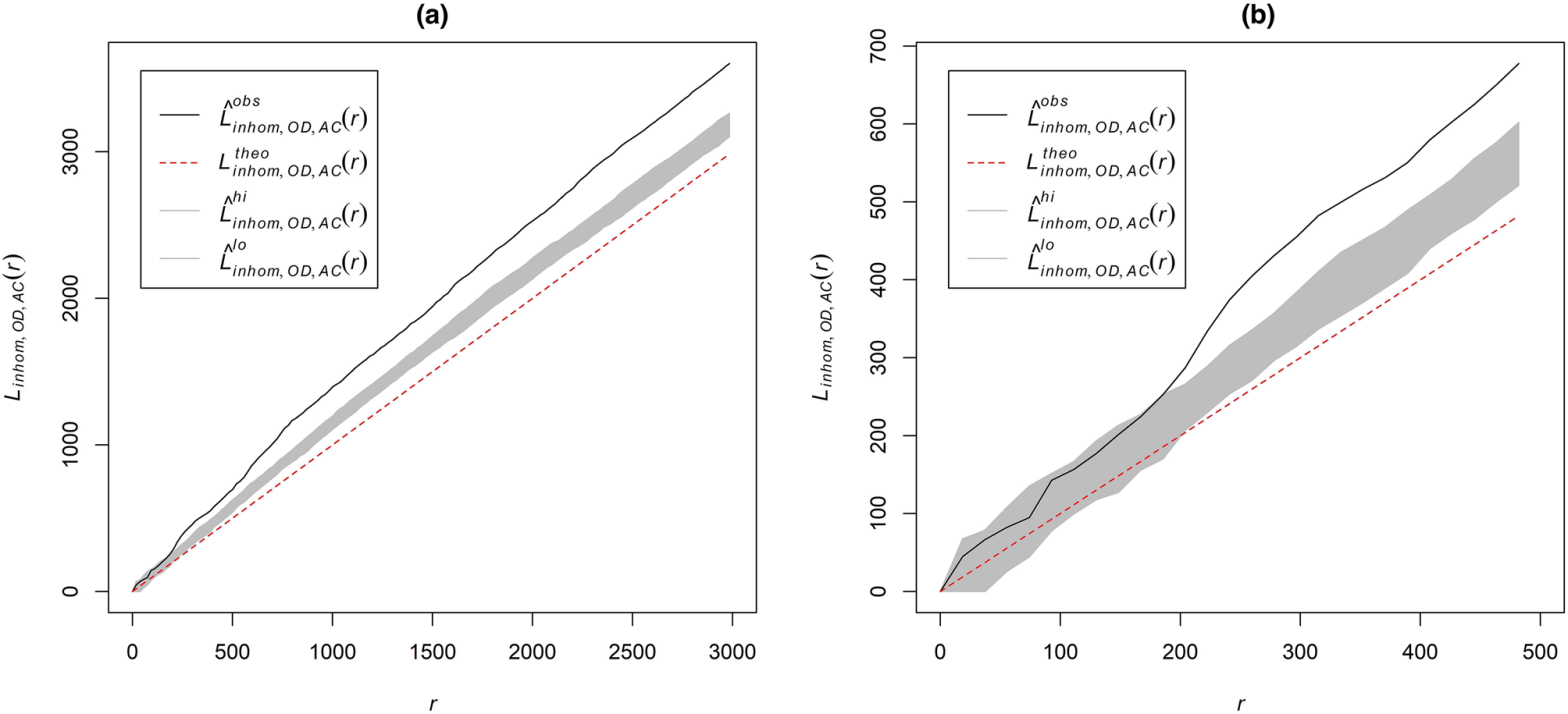
Characterizing cross point pattern between OOEs and animal complains related 311 calls, 2013. (**a**) Graph view with 3000 m maximum distance; (**b**) Graph view with 500 m maximum distance. Figures produced using the software R^25^.

To assess the integrity of the analysis, we tested results using distance buffers from 1 to 3000 m with a 1-m interval as shown in Fig. 1a, however we only focus on spatial dependence with a distance less than 500 m as shown in Fig. 1b, since the apparent spatial influence typically attenuated at a steep rate with distance^24^.

Figure 1 shows an example of the inhomogeneous cross-*K*-function between OOEs and animal complaint 311 calls in the year 2013. The shaded area is a 99% level confidence envelope under the hypothesis of CSR. By focusing on the Fig. 1b, we observed OOEs and 311 calls initially exists a random spatial pattern but exhibits clustering at 200-m distance thereafter. Supplementary Figs. S1 and S2 provides examples of dispersed and random spatial point patterns.

Figure 1 illustrates one 311 request type for one year. We generated 210 cross-point pattern analyses, corresponding to 21 request categories evaluated annually over the 10 year study period. Table 1 summarizes all annual pairwise comparisons across variety 311 types for the time period 2008–2017.

**Table 1:** Temporal trend of pairwise spatial dependences between OOE and 311 categories, annually, 2008–2017.

Full name	Abbr	Year (20–)									
		08	09	10	11	12	13	14	15	16	17
Abandoned vehicles	AV	C**	C**	C**	C**	C**	C**	C**	C**	C**	C**
Animal complains	AC	C*	C*	C*	C*	C**	C*	C*	C**	C**	C**
Bike related items	BRI	R	R	N/A	R	R	D	R	R	R	R
Code violation	CV	N/A	C**	C**	C*	C**	C**	C**	C**	C**	C**
Fire hydrant	FH	N/A	N/A	N/A	R	R	R	R	R	R	C*
Graffiti	GRA	C*	C*	C*	C*	C*	R	R	R	R	R
Homeless advocacy	HA	N/A	N/A	R	R	R	R	R	R	C**	C**
Law enforcement	LE	C**	C**	C**	C**	C**	C**	C**	C**	C**	C**
Parking	PAR	R	R	D	R	C*	C*	C**	R	C**	C**
Public health	PH	C*	C**	C**	C**	C**	C**	C**	C**	C**	C**
Recreation and parks	RP	R	R	R	R	R	R	R	R	R	R
Recycling yard waste	RYW	C**	R	C*	C*	C**	C**	C**	C**	C**	C**
Refuse trash litter	RTL	C**	C**	C**	C**	C**	C**	C**	C**	C**	C**
Sidewalk curbs ramps	SCR	C*	C*	C**	C*	C*	C*	C**	C**	C**	C**
Snow ice removal	SIR	C*	C*	C**	C**	C*	C*	C**	C*	R	R
Street lighting	SL	C**	C**	C**	C**	C**	C**	C**	C**	C**	C**
Street maintenance	SM	C**	C**	C**	C**	C**	C**	C**	C**	C**	C**
Traffic signals	TS	R	R	C*	C*	C**	C**	C**	C**	C**	C**
Traffic signs	TS2	C**	C**	C**	C**	C*	C**	C**	C**	C**	C**
Trees	TREES	R	R	C*	C*	C**	C*	C**	C*	C**	C**
Water sewers drains	WSD	C*	C*	C**	C**	C**	C*	C**	C*	C**	C**

C** Clustering at short distance (< 100 m), C* clustering at a long distance (100–500 m), *R* random pattern, *D* dispersed pattern, *N/A* data not available.

From Table 1 we observe that for 311 calls like abandoned vehicles, law enforcement, refuse trash litter, street lighting, and street maintenance, they are consistently clustered with OOEs starting at short distances. Likewise, for categories animal complains, code violation, public health, sidewalk curbs ramps, traffic signs, and water sewers drains they are also persistently spatial clustered with OOEs but starting at a relatively longer distance. However, for categories like bike related items and recreation parks showed random spatial pattern with opioid overdose events across timepoints, thus they are not able to serve as indicators for opioid overdose dynamics.

### Neighborhood clusters based on 311 requests and socioeconomic status

**Figure 2:**
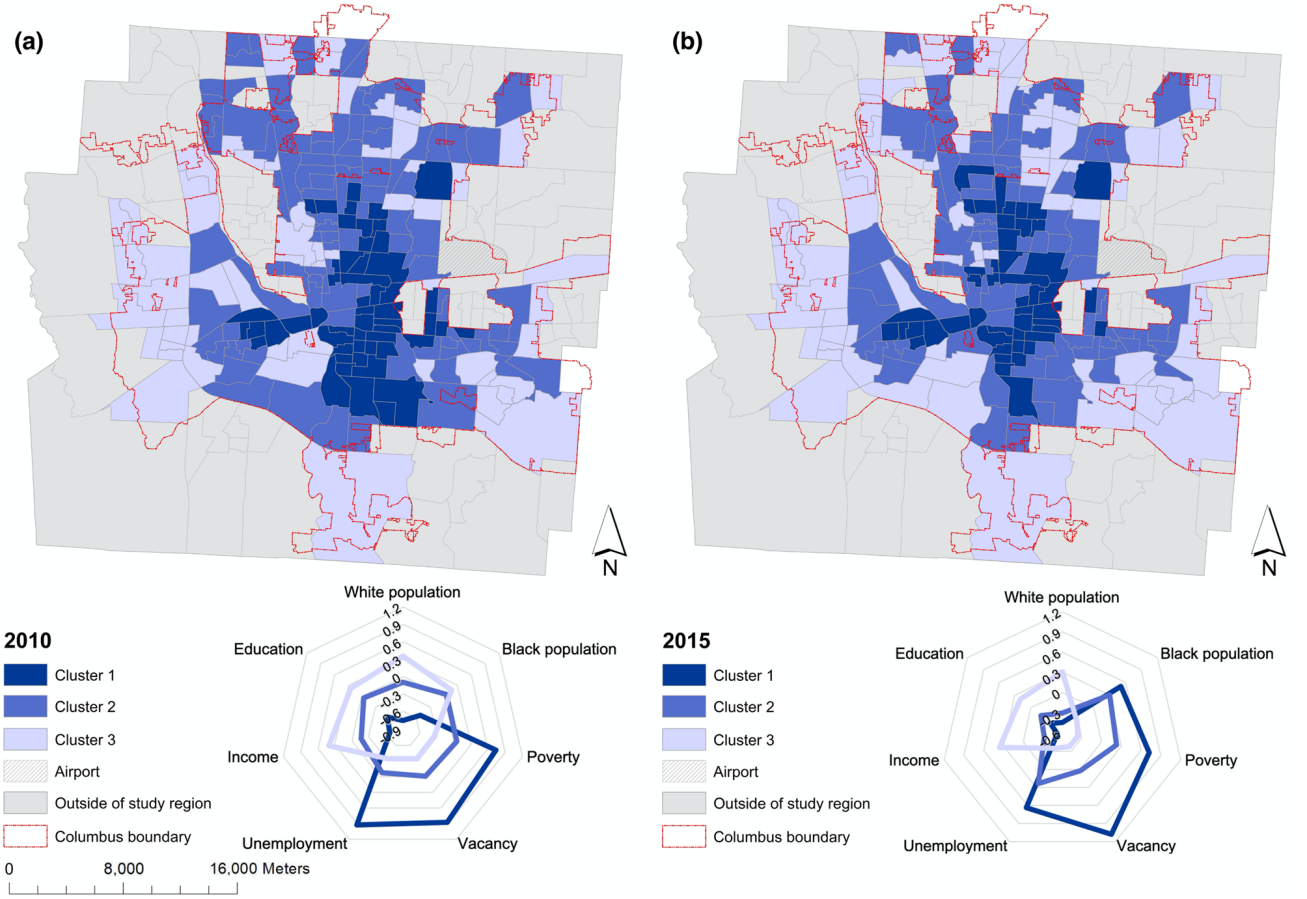
Spatial distribution and the socioeconomic profiles of the 311 clusters, Columbus, OH. (**a**) 2010; (**b**) 2015. Maps generated using the software ArcGIS Desktop^26^.

We employed an unsupervised K-means clustering analysis to generate three different neighborhood types based on the 311 categories. For comparability with 2010 and 2015 American Community Survey (ACS) data, we cluster census tracts based on 311 requests for two five-year periods, 2008–2012 and 2013–2017, using a frequency index as the measure. We match the first-5 years aggregated 311 data with 2010 ACS data and the second 5-year data corresponding to ACS 2015. We eliminate Bike Related Items, Fire Hydrant, and Homeless Advocacy for the first 5 years dataset due to the missing data records before 2012 (Table 1). Figure 2 shows the mapped neighborhood clusters and their socioeconomic profiles. All three clusters are different across all selected socio-economic variables (Supplementary Tables S1 and S2) for both periods (p < 0.001). Cluster 1 has the highest poverty, housing vacancy, unemployment rate, and the lowest education rates and income levels. Cluster 3 has high education rates and income levels with lowest unemployment, housing vacancy and poverty rates. Cluster 2 has a moderate socioeconomic profile relative to other two clusters. The spatial footprints and the socioeconomic profiles of the clusters are stable over time.

### Stability and robust indicators for OOEs

We assess the stability of the spatial associations between 311 categories and OOEs though analysis at a higher temporal resolution, using three-month periods for the years 2016 and 2017. From results in Table 2 we observed that for 311 categories like code violation, law enforcement, public health, and refuse trash litter associate with OOEs at short distances. Abandoned vehicles, animal complaints, recycling yard waste, sidewalks curbs ramps, street lighting, street maintenance, traffic signals, and traffic signs associate with OOEs, but over longer, varying distances. 311 categories such as bike related items, fire hydrant and recreation and parks show no spatial association with OOEs. The three-month based analysis shows a result consistent with analysis using a yearly temporal resolution.

**Table 2:** Temporal trend of pairwise spatial dependences between OOE and 311 categories, quarterly, 2016–2017.

Full name	Abbr	Q1	Q2	Q3	Q4	Q5	Q6	Q7	Q8
Abandoned vehicles	AV	C**	C**	C*	C**	C**	C**	C**	C**
Animal complains	AC	C*	C**	C**	C**	C*	C**	C**	C**
Bike related items	BRI	R	R	R	R	R	R	R	R
Code violation	CV	C**	C**	C**	C**	C**	C**	C**	C**
Fire hydrant	FH	R	R	R	R	R	R	R	R
Graffiti	GRA	R	C*	C*	R	R	C*	C*	C*
Homeless advocacy	HA	R	C*	C*	R	C*	C**	C**	C**
Law enforcement	LE	C**	C**	C**	C**	C**	C**	C**	C**
Parking	PAR	R	C*	C*	R	C*	C*	C*	C*
Public health	PH	C**	C**	C**	C**	C**	C**	C**	C**
Recreation and parks	RP	R	R	R	R	R	R	R	R
Recycling yard waste	RYW	C*	C**	C**	C**	C**	C**	C**	C*
Refuse trash litter	RTL	C**	C**	C**	C**	C**	C**	C**	C**
Sidewalk curbs ramps	SCR	C*	C*	C*	C*	C*	C*	C*	C*
Snow ice removal	SIR	R	N/A	N/A	R	R	N/A	N/A	R
Street lighting	SL	C*	C*	C**	C**	C**	C**	C**	C*
Street maintenance	SM	C**	C**	C**	C*	C**	C**	C**	C**
Traffic signals	TS	C*	C*	C*	C*	C*	C*	C*	C**
Traffic signs	TS2	C*	C*	C**	C**	C**	C**	C*	C*
Trees	TREES	R	C*	C*	C*	C*	C**	C**	C*
Water sewers drains	WSD	C**	C**	C*	C*	C*	C*	C*	R

C** Clustering at short distance (< 100 m), C* clustering at a long distance (100–500 m), *R* random pattern, *D* dispersed pattern. No ‘Snow Ice Removal’ calls reported from April to September for both years.

The neighborhoods comprising cluster 1 (Fig. 2) are most likely to be associated with socioeconomic distress^23^. Supplementary Fig. S3 shows the frequency index of 311 categories within cluster 1 for 2010 and 2015. We identify high frequency categories using a threshold of 50% of the maximum frequency index value (0.81 for 2010 and 0.84 for 2015).

Table 3 shows high frequency 311 types within distressed neighborhood cluster and 311 types that are spatially associated with OOEs in its time slot. We define robust 311 indicators as those with high relative frequency in distressed neighborhoods and spatially associate with OOE, consistently over time (both conditions met in 2010 and 2015). Based on these criteria, we identify abandoned vehicles, animal complaints, code violation, law enforcement, public health, refuse trash litter, street lighting, street maintenance, traffic signs, and water sewers drains as robust indicators.

**Table 3:** Comparison between spatial point pattern and SES clustering.

Time span	2008–2012		2013–2017	
	Spatially associated with OOEs	High frequency in distressed cluster	Spatially associated with OOEs	High frequency in distressed cluster
**Abandoned vehicles**	Yes	Yes	Yes	Yes
**Animal complains**	Yes	Yes	Yes	Yes
Bike related items	N/A	N/A	No	No
**Code violation**	Yes	Yes	Yes	Yes
Fire hydrant	N/A	N/A	No	No
Graffiti	Yes	No	No	No
Homeless advocacy	N/A	N/A	Mixed	No
**Law enforcement**	Yes	Yes	Yes	Yes
Parking	No	No	Mixed	No
**Public health**	Yes	Yes	Yes	Yes
Recreation and parks	No	No	No	No
Recycling yard waste	Mixed	Yes	Yes	Yes
**Refuse trash litter**	Yes	Yes	Yes	Yes
Sidewalk curbs ramps	Yes	No	Yes	Yes
Snow ice removal	Yes	No	Mixed	No
**Street lighting**	Yes	Yes	Yes	Yes
**Street maintenance**	Yes	Yes	Yes	Yes
Traffic signals	Mixed	No	Yes	No
**Traffic signs**	Yes	Yes	Yes	Yes
Trees	Mixed	Yes	Yes	Yes
**Water sewers drains**	Yes	Yes	Yes	Yes

Bold indicates robust surveillance indicators.

### Prediction of OOE hotspots

We assess the ability of the robust 311 indicators to predict OOE hotspots. We calculate the probability of the OOE occurrence surrounding 311 indicators with three buffer rings: 0–150 m, 150–300 m, and 300–500 m in 2016. We use these probability indexes with corresponding 311 calls in 2017 to predict the OOE spatial point pattern and aggregate the prediction points into census block group for a hotspot analysis. Using the Getis-Ord Gi* statistic^27^, we generate hotspots and coldspots at three confidence levels: 90%, 95%, 99%.

To measure the predictive accuracy of the 311 indicators, we use a confusion matrix^28^ to compare actual hotspots at the 95% confidence level and above with predicted hotspots at 95% confidence and above. Table 4 provides key parameters from the confusion matrix. Precision is the ratio of correctly predicted hotspots to the total predicted hotspots. Sensitivity is the ratio of correctly predicted hotspots to all actual significant hotspots. F1-score is the weighted average of precision and sensitivity; it is a general indicator of predictive accuracy that considers false positives and false negatives. With all three measures, values closer to unity indicate better performance.

**Table 4:** Predictive performance of 311 indicators for hotspots with 95% confidence or greater.

	Precision	Sensitivity	F1-score
Abandoned vehicle	0.88	0.79	0.83
Animal complains	0.79	0.52	0.62
Code violation	0.91	0.93	0.92
Law enforcement	0.89	0.63	0.73
Public health	0.84	0.94	0.89
Refuse trash litter	0.73	0.76	0.74
Street lighting	0.78	0.88	0.83
Street maintenance	0.56	0.47	0.51
Traffic signs	0.75	0.49	0.60
Water sewer drains	0.51	0.30	0.38

**Figure 3:**
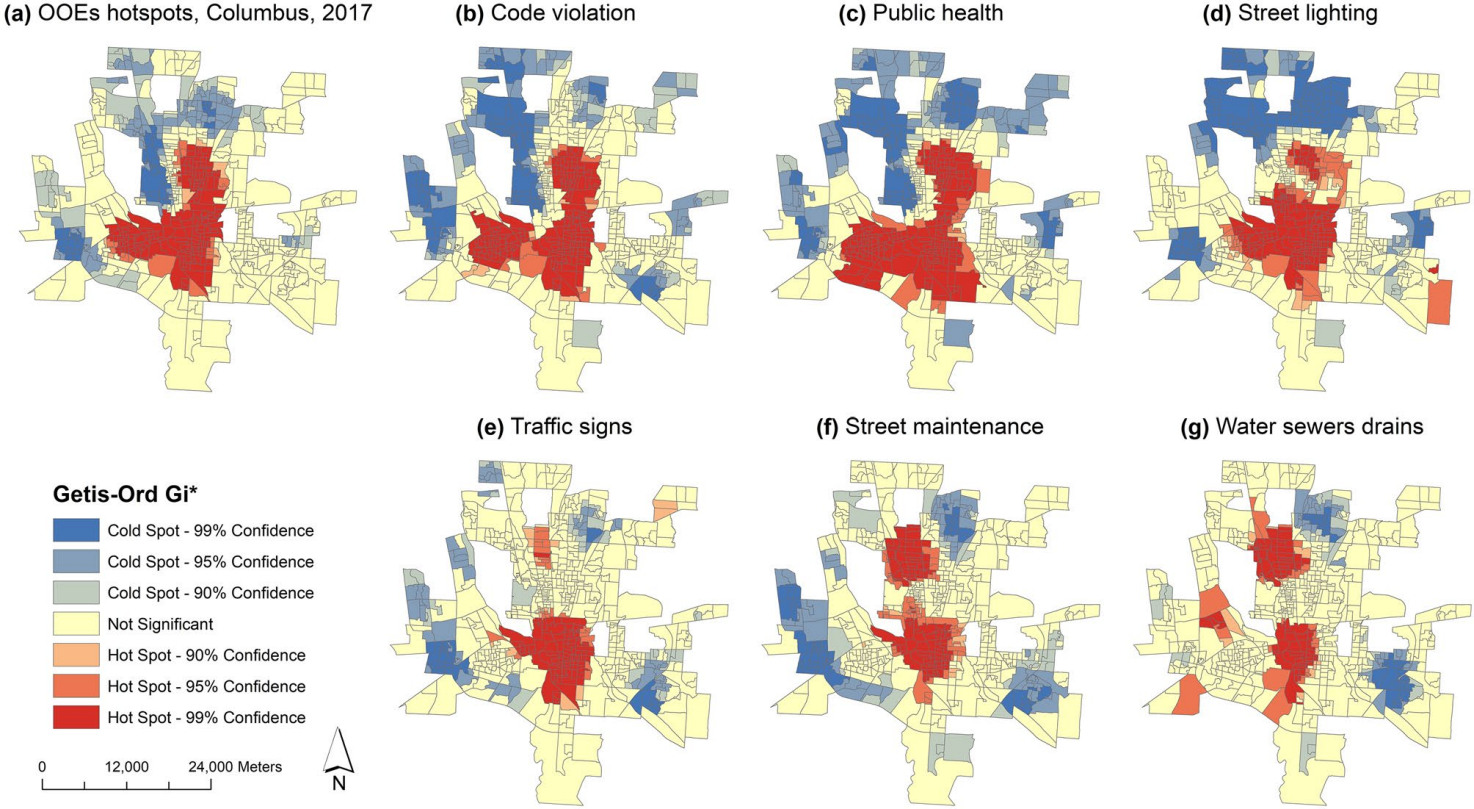
Spatial distribution of the OOE hot/cold spots in 2017. (**a**) actual hot/cold spots; (**b**) prediction from ‘Code violation’; (**c**) prediction from ‘Public health’; (**d**) prediction from ‘Street lighting’; (**e**) prediction from ‘Traffic signs’; (**f**) prediction from ‘Street maintenance’; (**g**) prediction from ‘Water sewers drains’. Maps generated using the software ArcGIS Desktop^26^.

We perform hotspot prediction and validation in the year 2017 when our most recent OOE data was collected. Figure 3 compares actual and predicted hot and cold spots for the year 2017. Figure 3a shows the actual spatial distribution of OOE hot spots and cold spots in Columbus, 2017. The remaining maps show the three most accurate predictors based on F1-score: code violation (Fig. 3b), public health (Fig. 3c) and street lighting (Fig. 3d). Figure 3 also shows the three most inaccurate predictions: traffic signs, street maintenance, and waters sewers drains in Fig. 3e–g, respectively. Prediction results from the remaining 311 categories can be found in Supplementary Fig. S4.

## Discussion and conclusion

Higher opioid overdose events rates in lower socioeconomic status neighborhoods support the view that its etiology is rooted in concentrated disadvantages, isolation and social upheaval. As the epidemic wages on, research that examines neighborhood-level data versus individual level data could provide insight into this significantly complex problem. The key objective of public health surveillance is to provide information to guide interventions^29^, and our analysis revealed 311 data can be used to characterize the level of neighborhood distress and further serves as an indicator for opioid crisis. According to the National Institute on Drug Addiction^3^, drug overdose data for the year 2019 will be updated in early 2021, and it tends to be aggregated by state level. Such long lag time for outcome data may not be very helpful for each county to plan responses to the opioid epidemic. In this paper, we demonstrate that 311 data can serve as a readily available alternative for real-time health outcome data for monitoring or predicting the spread of the opioid overdose crisis. These data can also shed lights on the social and neighborhood determinants of the opioid crisis, as a guide for local communities to target the issues most associated with overdose deaths and emergencies.

In our analysis, service requests about abandoned vehicles, animal complaints, code violation, law enforcement, public health, recycling yard waste, refuse trash litter, street lighting, street maintenance, traffic signs, trees, and water sewers drains are closely associated with census indicators for neighborhood distress, and 10 of 12 (except recycling yard waste and trees) display robust spatial clustering with opioid overdose events. Service request/complaints related to bikes, fire hydrants, homeless advocacy, parking, and recreation parks not only fail to connect with social-neighborhood distress, but also are not spatially correlated with opioid overdose events. We conclude that abandoned vehicles, animal complaints, code violation, law enforcement, public health, refuse trash litter, street lighting, street maintenance, traffic signs and water sewer drains can serve as robust indicators for opioid dynamics. We show that several categories of 311 requests are good predictors OOE hotspots individually, even though the predictive performance varies among the categories (as shown in Fig. 3 and Supplementary Fig. S4).

Neighborhood disadvantage, distress and disorder affect an individual’s physical and psychological health. Visible signs of social disorder include disputes among neighbors, noise, crime activities^23^, the presence of anti-social behavior^30^ and using drugs and alcohol^31^. Physical disorder refers to the general unsatisfied neighborhood appearance including litter, vandalism, disrepair or abandoned properties^32^, food security or sanitation issues^33^, poorly maintained facilities or unsatisfied housing quality, and so on.

The 311 indicators we identified in our analysis track social and physical urban disorders. Table 5 classifies the robust 311 indicators along the dimensions of social and physical disorder, and provides the official definitions used to classify individual 311 requests into different types. These definitions suggest measurable variables that can be explored in focused research, or potentially be targets for improving neighborhood issues associated with opioid crisis.

**Table 5:** Classification of robust 311 indicators by social versus physical disorder.

Dimension	311 request type	Definition
Social disorder	Abandoned vehicles	Abandoned vehicle which has been parked on a street for more than 24 h
	Animal complains	Dangerous, dead, sick, or unsanitary animals in the public
	Code violation	Unintended object on private property (e.g. high grass or vehicle) and dangerous/ disrepair structure
	Law enforcement	Anti-social/ illegal behavior (e.g. narcotics, speeding, noise, crime etc.)
	Public health	Pest management, food security and unsanitary conditions due to animals
Physical disorder	Refuse trash litter	Trash container needs repaired, replaced and serviced
	Street lighting	Streetlight needs repaired
	Street maintenance	Pothole, street, and alley need repaired
	Traffic signs	Repair, replace or removal of an existing traffic sign or installation of a new one
	Water sewers drains	Issue with the water and sewer

The 311 taxonomy definitions in Table 5 also points to a limitation: these definitions are inconsistent across communities, or across time. This limits the potential to compare 311 indicators across cities, and even within a single city across time if the city does not maintain consistent definitions. A well-established national data standard for 311 categories can be a potential solution to reconcile the semantic heterogeneity of 311 data. It may also be possible to develop concept mappings that can translate data between the categories in different communities. Communities should also maintain metadata on these classifications, including translation pathways between categories when they change.

There has been extensive attention to the supply that contributes to the present opioid crisis^34^. Without denying that opioid availability might serve as a catalyst for the crisis, we argue that such public health crisis is jointly linked to the ‘demand side’ of substance misuse—the physical and psychological needs for opioids. A variety of structural factors such as poverty, lack of opportunity, and substandard living and working conditions may lead individuals’ hopelessness and despair^35^. Individuals living in an unsatisfactory socioeconomic neighborhood were more likely to develop such disease of despair, which further increased the risk for non-medical use of opioids^6^.

Although quantitative assessment of how socio-neighborhood distress affects the opioid crisis can be challenging, it can also be beneficial in guiding policy responses^18^. Likely due to these challenges, few opioid-related research studies explore the effect of social distress at the neighborhood level. Our research approach of using 311 service calls as indicators, however, offers one possible way to examine the spatial associations between social-neighborhood distress and opioid overdose, and further serve as a low-cost but high-resolution surveillance indicator for opioid crisis monitoring. We use opioid overdose hotspot prediction as a case study, however our research framework developed here are applicable to more social neighborhood determinants of health research such as how neighborhood distress indicators spatially correlate with other substance abuses.

There are several facets for potential improvement or further exploration. First, we tried different spatial aggregation levels (census block group, census tract, and zip code) for 311 data but found no significant density of 311 requests at block group level, especially for the first few years. To ensure a statistically-significant number of 311 requests for each areal unit, we aggregated our 311 requests at census tract level; however, since US Census American Community Survey (ACS) or other socioeconomic data may be available at the block group level or similar geography, a finer spatial resolution could be achieved if the number of 311 requests is large enough. Second, we use k-means clustering as a basic approach to group neighborhoods based on 311 data. Although our results are clear and stable, other methods may provide a more nuanced characterization of differences among neighborhoods based on these requests. Third, our selection of demographic and socioeconomic variables is largely based on a review of previous literature. Required is further investigation of the socioeconomic and neighborhood covariates with opioid use disorder.

## Methods

### Data

We obtained individual-level opioid overdose events from electronic patient care records obtained from the Columbus Fire Department. Criteria for identifying an OOE include: (1) naloxone was administered by emergency medical services (EMS)/law enforcement/fire fighter and/or (2) impression or chief complaint was one of the following: opioid related disorders, substance abuse, drug abuse, poisoning/drug ingestion, cocaine related disorders, altered mental state, cardiac arrest. We carried out a geocoding process for all the scene addresses of selected paramedic runs and we successfully matched more than 75% of the addresses. Based on attributes included in the EMS data, 73.5% of OOEs occurred in a residential location. Each OOE was resolved to the date that the event transpired, with a total number of 10,426 records between 1/1/2008 and 12/31/2017. For this overall sample, the majority of events occurred in 25–35 years old (32.39%) white (58.65%) men (59%), a descriptive statistic for individual level demographic profile can be found in Supplementary Table S5.

311 is a non-emergency request system that allows residents to report low priority issues that require governmental attention. Many cities in the United States support these services. Columbus 311 system allows anyone to report an issue via email, webpage or a smartphone app. Georeferencing for these issues involves automated location referencing in the smartphone app, and users reporting street addresses or closest intersection. For most cities including Columbus, the accurate street address or geographical coordinates of the reported issue are recorded into a database^36,37^. The City of Columbus Department of Neighborhoods maintains a retrospective geocoded dataset of 311 service requests that contains 21 different categories of non-emergency public issues. Each record provides some basic information about the request, such as the spatial location of the request, the nature of the request service in different level of categories, when this request was received and when it was completed. We acquired all service requests received from January 1, 2008 to December 31, 2017; this comprised 800,511 records. Supplementary Table S3 shows the numbers of 311 requests over time along with OOEs from the EMS data. Also note the number of service categories changed over time but was stable from 2011–2017. The dataset covers the entire administrative boundary of city Columbus, Ohio.

We use 311 data as proxies for socioeconomic conditions of urban neighborhoods^37^, we compare these data with administrative data to census data to confirm that the types of 311 requests that are spatially associated with OOEs also associate with other indicators of socioeconomic distress. We use American Community Survey (ACS) 5-year estimates data for the years of 2010 and 2015. The 2010 ACS 5-year estimates data was not available at the level of block group; therefore, we use data at the census tract level which is also the finest spatial aggregation level for both years. Due to the nature of boundary mismatching between city of Columbus and census data, we only use census tracts which fall within the administrative boundary of Columbus (as shown in Fig. 2). More specifically, a tract with more than half of its area within the city’s boundary is our study area. The census tract boundaries are consistent for both years and there were total number of 204 census tracts for each year. We select multiple variables and eventually integrate them as seven major socio-economic indicators in race, economics, education, and housing (Supplementary Table S4). All variables were standardized as z-scores to have a consistent measurement scale.

### Analysis workflow

Supplementary Fig. S5 illustrates the workflow in our analysis, highlighting the pathways corresponding to the following research objectives. Objective 1: discover the spatial association between OOEs and 311 service requests types. Objective 2: identify the types of 311 requests that characterize neighborhoods with socioeconomic distress. Objective 3 assess the stability of the spatial associations between 311 requests and OOEs. Objective 4: evaluate the performance of the robust 311 indicators in predicting overdose hotspots.

### Inhomogeneous cross-K-function

Derived from Ripley's *K*-function^38^, a well-known second order statistic to describe the spatial point patterns, we employ an inhomogeneous cross-*K*-function^39,40^ to test the spatial point pattern interactions between OOEs and 311 calls. This method counts the expected number of one type of points within a given distance of another type of points, adjusted for spatially varying intensity.

Given a non-stationary multivariate spatial point process $S$ with $p$ sub-processes, $S=\left\{{S}_{1},\dots , {S}_{p}\right\}=\left\{\left\{{S}_{11},\dots ,{S}_{1N1}\right\},\dots ,\left\{{S}_{p1},\dots ,{S}_{pNp}\right\}\right\}$ with spatially varying intensity function ${\lambda }_{i}(s)$ for subprocess $i$, the inhomogeneous cross-*K*-function between sub-process ${S}_{n}$ and ${S}_{m}$ is denoted as:

1 $${K}_{inhom}\left(m,n;h\right)=\frac{1}{\left|R\right|} \sum_{i=1}^{{N}_{m}}\sum_{j=1}^{{N}_{n}}\frac{I\left({d}_{ij}\le h\right)}{{\lambda }_{m}\left({s}_{m,i}\right){\lambda }_{n}\left({s}_{n,j}\right)}$$

where $\left|R\right|$ denotes the area of study region $R$; ${N}_{m}$, ${N}_{n}$ are the total number of points for sub-processes ${S}_{m}$ and ${S}_{n}$ respectively; ${d}_{ij}$ denotes the distance between point ${s}_{m,i}$ in sub-process ${S}_{m}$ and point ${s}_{n,j}$ in sub-process ${S}_{n}$; $I\left({d}_{ij}\le h\right)$ equals to 1 if ${d}_{ij}\le h$ and 0 otherwise. ${\lambda }_{m}$ and ${\lambda }_{n}$ are non-parametric kernel estimations of the intensity surface for sub-process ${S}_{m}$ and ${S}_{n}$ respectively^41^.

To detect the departure from independence, we perform 99 Monte Carlo simulations and generate a 99% confidence envelope for each assessment^42^. We also transformed our *K*-function result into an equivalent *L*-function format for better visual representation by taking:

2 $$L\left(r\right)= \sqrt{K(r)/\pi }$$

Spatial analysis and its significance test were completed by using *spatstat* package^43^ in software R^25^.

### K-means clustering and analysis of variance

We use K-means clustering to determine neighborhood types based on 311 requests^37,44^. We aggregate 5-year (2008–2012) 311 calls into 2010 ACS census tract boundary by category counts, similarly with second 5-year (2013–2017) and 2015 ACS census geography. We convert the summary counts into standardized rates. This creates a unique 311-pattern signature for each census tract, reflecting the unique mix of request types characterizing the neighborhood, we use ANOVA to test for differences among neighborhood clusters based on socioeconomic attributes.

### Point based simulation and hotspot analysis

Unlike other regular measured space–time point processes such as land surface vegetation index^45^ or road traffic^46^, public health events like OOEs tend to be sparser in both space and time. Thus, they cannot be simply aggregated into a small geographical boundary due to the number of event counts at the minimum geographical unit may not be large enough to have statistical power^47^. So, we propose a point-based prediction method to address such a scenario that is unsuitable for conventional statistical modelling.

We count the total number of sub-point process ${S}_{m}$ within a given distance interval ${h}_{k+1}- {h}_{k}$ of another type sub-point process ${S}_{n}$ at time $t$, estimated as:

3 $$O\left({h}_{k}\right)= \left\{\begin{array}{c}\sum_{i=1}^{{N}_{m}}\sum_{j=1}^{{N}_{n}}I\left({d}_{ij}\le {h}_{k}\right), k=1\\ \sum_{i=1}^{{N}_{m}}\sum_{j=1}^{{N}_{n}}I\left({h}_{k-1}{\le d}_{ij}\le {h}_{k}\right), k \ge 2\end{array}\right.$$

We further use $O\left({h}_{k}\right)$ to calculate how likely a point process ${S}_{m}$ will occur within a given distance interval ${h}_{k}- {h}_{k-1}$ of another type sub-point process ${S}_{n}$ estimated as:

4 $$\lambda \left({h}_{k}\right)= \frac{O\left({h}_{k}\right)}{\sum_{1}^{k}O\left({h}_{k}\right)}$$

We use $\lambda \left({h}_{k}\right)$ together with the observed point process ${S}_{m}$ at time $t+1$ to estimate the unknown sub-point process ${\widehat{s}}_{n}$. We randomly generate points for each point in ${S}_{m}$ within its buffer ring region ${h}_{k}- {h}_{k-1}$ with probability $\lambda \left({h}_{k}\right)$. For simplicity we only set up four different distance thresholds for later simulation: *h*_1_ = 0 m, *h*_2_ = 150 m, *h*_3_ = 300 m, and *h*_4_ = 500 m.

We aggregate both real point pattern and estimate point pattern into census block group level. We use a hotspot analysis to calculate the Getis-Ord Gi* statistic^27^, the prediction accuracies for 311 categories are further calculated.

### Ethics statement

This study was approved by The Ohio State University Biomedical Sciences Institutional Review Board (IRB) deemed to pose only minimal risk to human subjects (Approval # 2017H0220). The City of Columbus Division of Fire Ethics Committee also approved this study. Both the IRB and the Ethics Committee waived the need to obtain informed consent as the data is routinely collected for healthcare purposes, and attempts to contact study participants would create high risk for exposing their involvement in a potentially sensitive healthcare issue (opioid overdose). All methods in this research were performed in accordance with the relevant guidelines and regulations.

## Supplementary information

Supplementary Information.

## Data Availability

The datasets generated and analyzed during the current study are not publicly available due to privacy restrictions of sensitive data.
