# Supplementary material for: 311 service requests as indicators of neighborhood distress and opioid use disorder
Source: Sci Rep. 2020 Nov 11;10:19579. doi: 10.1038/s41598-020-76685-z (PMC7658248; doi:10.1038/s41598-020-76685-z)
Supplement: Supplementary file 1 — Supplementary Information. [file 41598_2020_76685_MOESM1_ESM.docx]

**Appendix**

**Supplementary Document for “311 service requests as indicators of neighborhood distress and opioid use disorder”, Scientific Reports**

Yuchen Li^1,2^, Ayaz Hyder^3^, Lauren T. Southerland^4^, Gretchen C. Hammond^5^, Adam Porr^2^ and Harvey J. Miller*^1,2^

^1^Department of Geography, The Ohio State University

^2^Center for Urban and Regional Analysis, The Ohio State University

^3^College of Public Health, The Ohio State University

^4^Wexner Medical Center, The Ohio State University

^5^Mighty Crow Media, Columbus, Ohio, USA

*Corresponding author: [miller.81@osu.edu](mailto:miller.81@osu.edu)


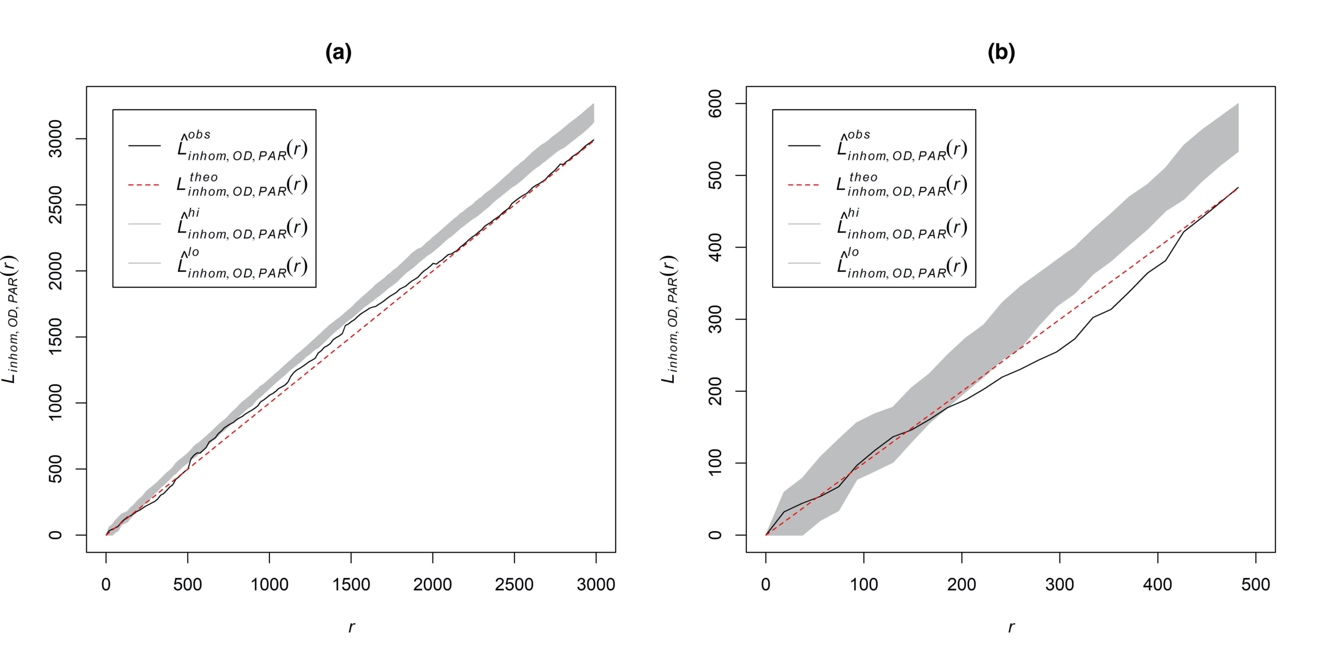


**Figure S1.** Characterizing cross point pattern between OOEs and parking related 311 calls, 2010. **(a)** Graph view with 3000 meters maximum distance; **(b)** Graph view with 500 meters maximum distance. Figures produced using the software R ^1^.

Supplementary Figure S1(a) shows the cross-point pattern within 3000-meter distance. Supplementary Figure S1(b) shows OOEs and parking related 311 calls initially exists a random spatial pattern but start dispersing at 160-meter distance thereafter.


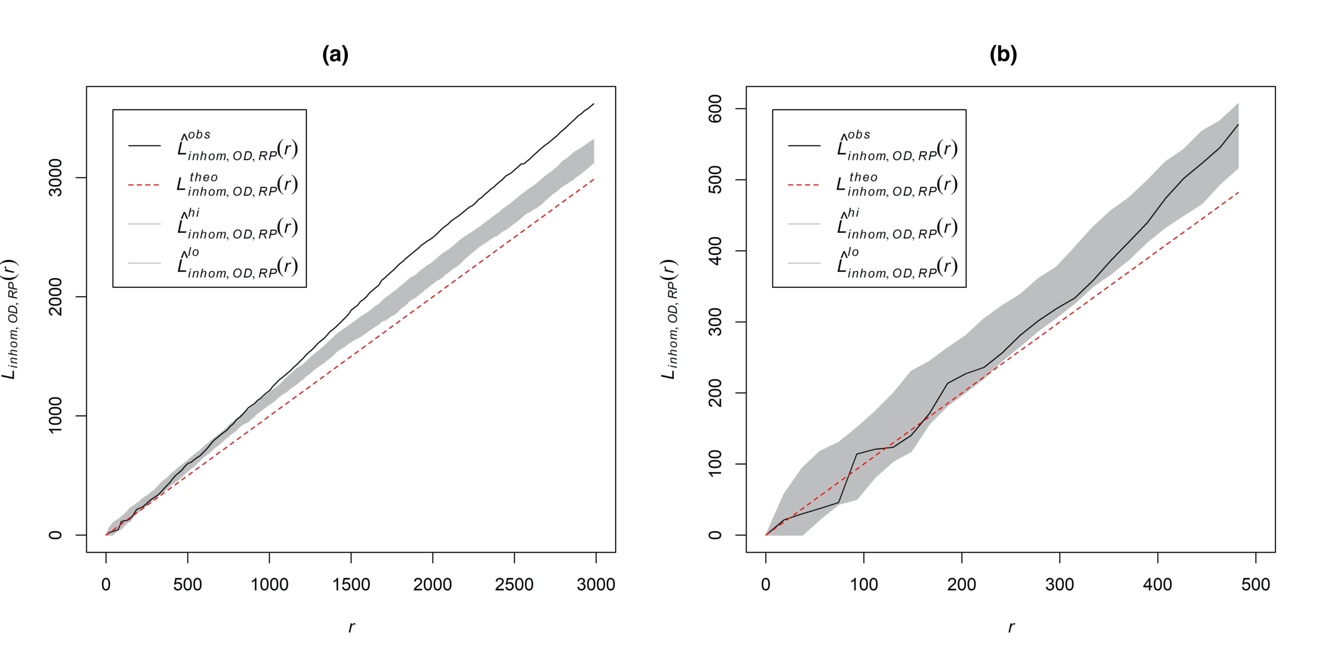


**Figure S2.** Characterizing cross point pattern between OOEs and recreation and parks related 311 calls, 2015. **(a)** Graph view with 3000 meters maximum distance; **(b)** Graph view with 500 meters maximum distance. Figures produced using the software R ^1^.

Supplementary Figure S2(a) shows the cross-point pattern within 3000-meter distance. Supplementary Figure S2(b) shows OOEs and recreation and parks related 311 calls exists a random spatial pattern within 500-meter distance.

﻿We have 21 types of 311 calls and conducted inhomogeneous cross-k-function Monte Carlo test 18 times (2008-2017 annually plus 2016-2017 quarterly) for each 311 categories. Therefore, our results comprise 378 graphs. Due to space limitations, we do not show all graphs. Table 1 and Table 2 in the main text summarize the results.

**Figure S3.** Frequency index of 311 categories within cluster 1, 2010 and 2015. Note: ‘bike related items’, ‘fire hydrant’, and ‘homeless advocacy’ not included in 2010 clustering.


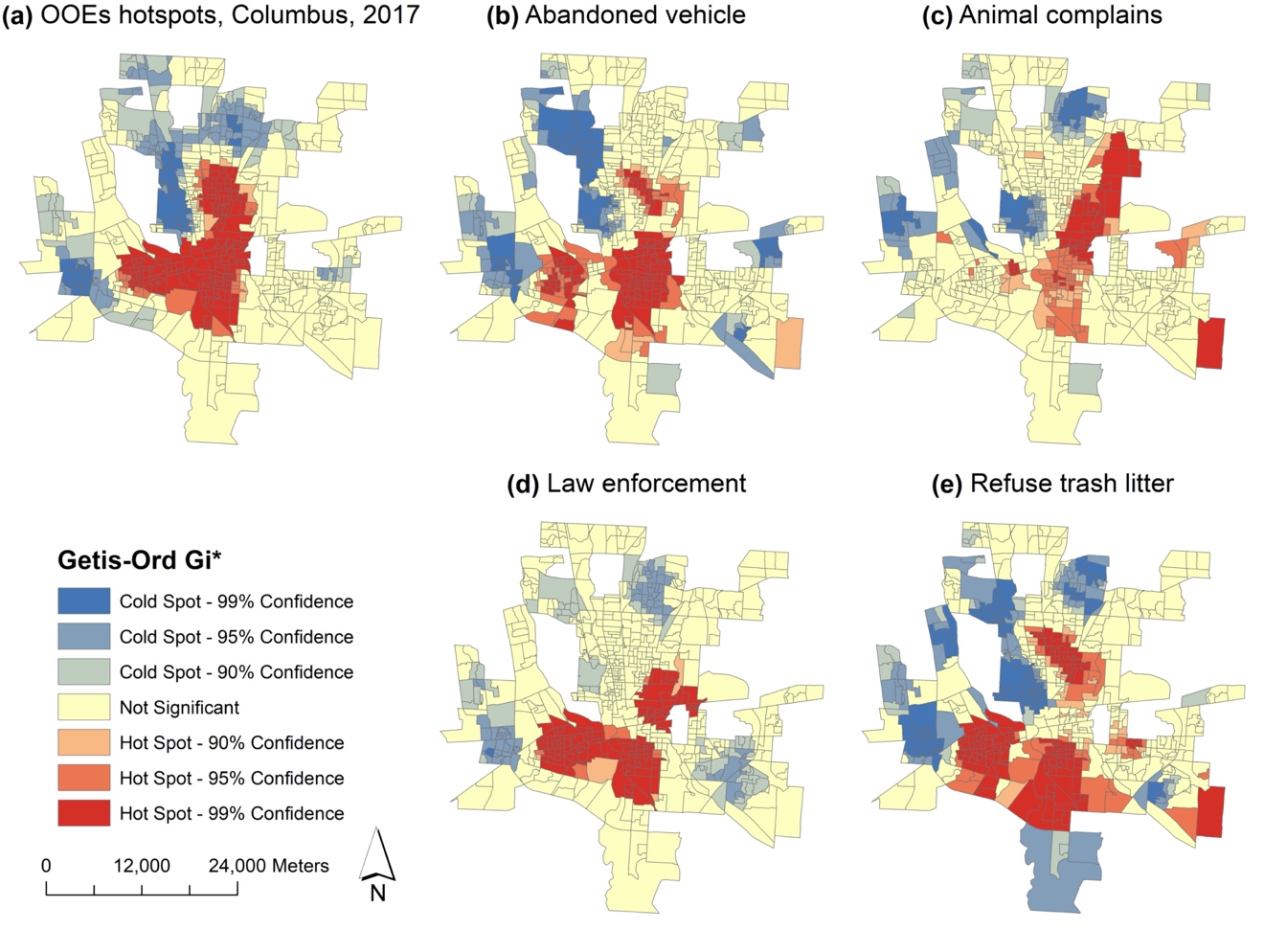


**Figure S4.** Spatial distribution of the OOE hot/cold spots in 2017. **(a)** actual hot/cold spots; **(b)** prediction from ‘Abandoned vehicle’ (F1-score 0.83); **(c)** prediction from ‘Animal complains’ (F1-score 0.62); **(d)** prediction from ‘Law enforcement’ (F1-score 0.73); **(e)** prediction from ‘Refuse trash litter’ (F1-score 0.74). Maps generated using the software ArcGIS Desktop ^2^.


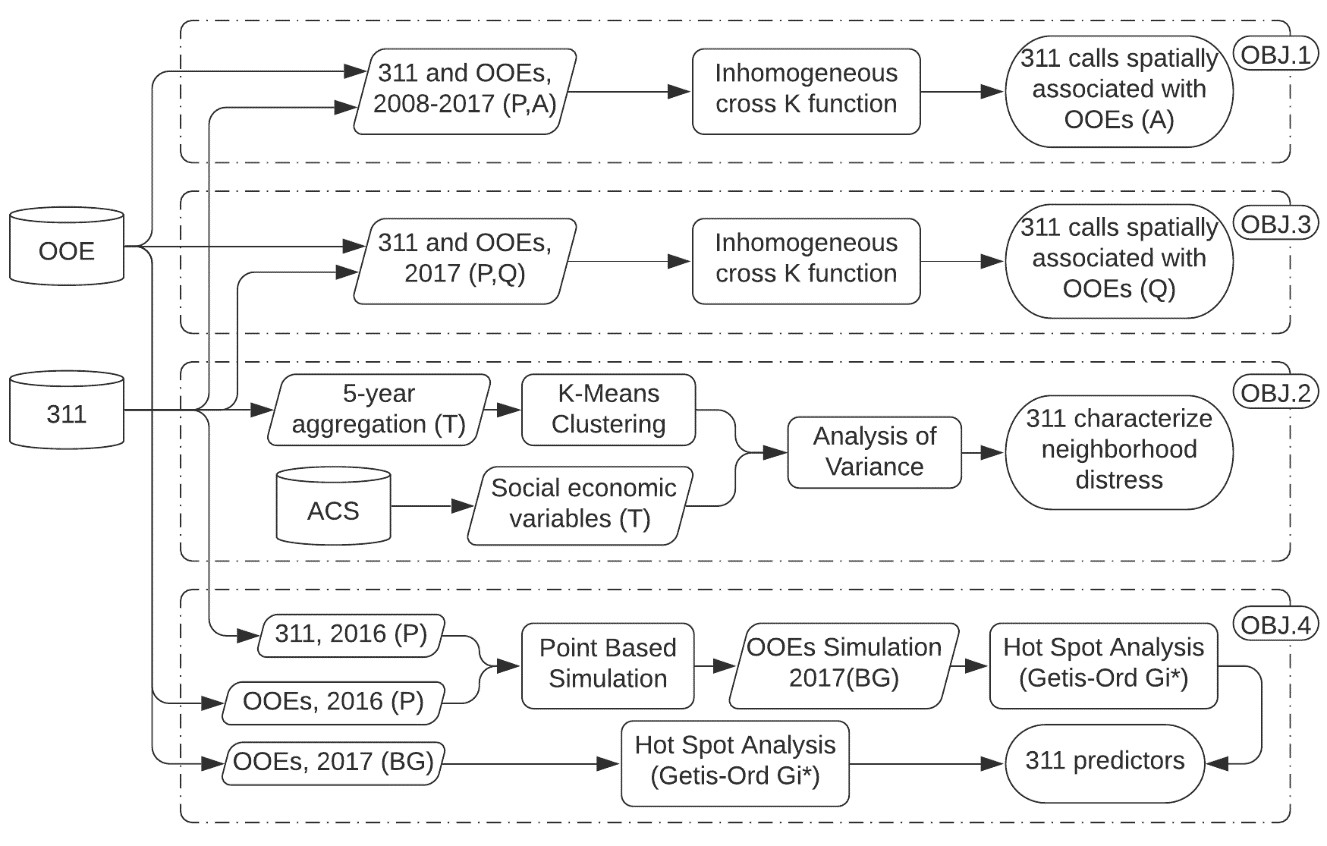


**Figure S5.** Analysis workflow. Note: P = point level; T = census tract level; BG = census block group level; A = Annually; Q = Quarterly.

Objective 1: discover the spatial association between OOEs and 311 service requests types. Objective 2: identify the types of 311 requests that characterize neighborhoods with socioeconomic distress. Objective 3 assess the stability of the spatial associations between 311 requests and OOEs. Objective 4: evaluate the performance of the robust 311 indicators in predicting overdose hotspots.

|  | | Sum of Squares | df | Mean Square | F | Sig. |
| --- | --- | --- | --- | --- | --- | --- |
| white population | Between Groups | 47.686 | 2 | 23.843 | 28.287 | .000 |
|  | Within Groups | 232.642 | 276 | .843 |  |  |
|  | Total | 280.329 | 278 |  |  |  |
| black population | Between Groups | 18.084 | 2 | 9.042 | 9.495 | .000 |
|  | Within Groups | 262.837 | 276 | .952 |  |  |
|  | Total | 280.921 | 278 |  |  |  |
| poverty | Between Groups | 44.668 | 2 | 22.334 | 27.423 | .000 |
|  | Within Groups | 224.779 | 276 | .814 |  |  |
|  | Total | 269.448 | 278 |  |  |  |
| vacancy | Between Groups | 54.651 | 2 | 27.325 | 33.286 | .000 |
|  | Within Groups | 226.575 | 276 | .821 |  |  |
|  | Total | 281.225 | 278 |  |  |  |
| unemployment | Between Groups | 60.204 | 2 | 30.102 | 41.461 | .000 |
|  | Within Groups | 200.386 | 276 | .726 |  |  |
|  | Total | 260.590 | 278 |  |  |  |
| income | Between Groups | 46.484 | 2 | 23.242 | 27.729 | .000 |
|  | Within Groups | 231.337 | 276 | .838 |  |  |
|  | Total | 277.821 | 278 |  |  |  |
| education | Between Groups | 24.232 | 2 | 12.116 | 13.176 | .000 |
|  | Within Groups | 253.786 | 276 | .920 |  |  |
|  | Total | 278.018 | 278 |  |  |  |

**Table S1.** Analysis of variance (ANOVA) table for 2010 k-means clustering.

|  | | Sum of Squares | df | Mean Square | F | Sig. |
| --- | --- | --- | --- | --- | --- | --- |
| white population | Between Groups | 31.740 | 2 | 15.870 | 17.667 | .000 |
|  | Within Groups | 247.027 | 275 | .898 |  |  |
|  | Total | 278.767 | 277 |  |  |  |
| black population | Between Groups | 35.057 | 2 | 17.529 | 19.797 | .000 |
|  | Within Groups | 243.494 | 275 | .885 |  |  |
|  | Total | 278.551 | 277 |  |  |  |
| poverty | Between Groups | 49.104 | 2 | 24.552 | 30.671 | .000 |
|  | Within Groups | 220.134 | 275 | .800 |  |  |
|  | Total | 269.239 | 277 |  |  |  |
| vacancy | Between Groups | 74.840 | 2 | 37.420 | 51.164 | .000 |
|  | Within Groups | 201.128 | 275 | .731 |  |  |
|  | Total | 275.968 | 277 |  |  |  |
| unemployment | Between Groups | 40.776 | 2 | 20.388 | 25.826 | .000 |
|  | Within Groups | 217.091 | 275 | .789 |  |  |
|  | Total | 257.866 | 277 |  |  |  |
| income | Between Groups | 39.903 | 2 | 19.951 | 22.965 | .000 |
|  | Within Groups | 238.917 | 275 | .869 |  |  |
|  | Total | 278.819 | 277 |  |  |  |
| education | Between Groups | 15.797 | 2 | 7.898 | 8.384 | .000 |
|  | Within Groups | 259.078 | 275 | .942 |  |  |
|  | Total | 274.874 | 277 |  |  |  |

**Table S2.** Analysis of variance (ANOVA) table for 2015 k-means clustering.

|  | 311 Service Calls | | Opioid Overdose |
| --- | --- | --- | --- |
| Year | # Categories | Total Requests | Total Events |
| 2008 | 17 | 53,957 | 413 |
| 2009 | 19 | 46,075 | 614 |
| 2010 | 19 | 69,165 | 747 |
| 2011 | 21 | 69,079 | 843 |
| 2012 | 21 | 65,572 | 882 |
| 2013 | 21 | 79,088 | 864 |
| 2014 | 21 | 101,133 | 920 |
| 2015 | 21 | 102,649 | 1,097 |
| 2016 | 21 | 102,866 | 1,463 |
| 2017 | 21 | 110,927 | 2,583 |
| Total |  | 800,511 | 10,426 |

**Table S3.** 311 and opioid overdose event counts for Columbus

| Variables | Interpretation |
| --- | --- |
| White population | % white population |
| Black population | % black/African American population |
| Unemployment | % persons 16+ years old who are in the civilian labor force and unemployed |
| Poverty | % total persons below the poverty level last year |
| Income | median household income |
| Vacancy | % total vacant year-round housing units |
| Education | % persons 25-64 years old with bachelor’s degree or higher |

**Table S4.** Socio-economic variable list

| Demographic Variable | Number of OOEs (%) |
| --- | --- |
| **Age** |  |
| < 18 | 188 (1.8%) |
| 18 - 24 | 1,498 (14.37%) |
| 25 - 34 | 3,377 (32.39%) |
| 35 - 44 | 2,224 (21.33%) |
| 45 - 54 | 1,753 (16.81%) |
| 55 - 64 | 984 (9.44%) |
| > 64 | 402 (3.86%) |
| Total | 10,426 (100%) |
| **Sex** |  |
| Female | 4,272 (40.97%) |
| Male | 6,151 (59%) |
| Unknown | 3 (0.03%) |
| Total | 10,426 (100%) |
| **Race** |  |
| Asian | 12 (0.12%) |
| Black | 1,658 (15.9%) |
| Hispanic | 110 (1.06%) |
| Other | 142 (1.36%) |
| Unknown | 2,389 (22.91%) |
| White | 6,115 (58.65%) |
| Total | 10,426 (100%) |

**Table S5.** Descriptive statistics for EMS runs data collected from 2008-2017

**References**

1. R Core Team. R: A language and environment for statistical computing. R Foundation for Statistical Computing, Vienna, Austria. URL https://www.R-project.org/ (2018).

2. ESRI. ArcGIS Desktop: Release 10.2, Redlands, CA: Environmental Systems Research Institute, USA. URL https://desktop.arcgis.com/ (2014).
